# Autophagy and Autophagy-Related Diseases: A Review

**DOI:** 10.3390/ijms21238974

**Published:** 2020-11-26

**Authors:** Tadashi Ichimiya, Tsukasa Yamakawa, Takehiro Hirano, Yoshihiro Yokoyama, Yuki Hayashi, Daisuke Hirayama, Kohei Wagatsuma, Takao Itoi, Hiroshi Nakase

**Affiliations:** 1 Department of Gastroenterology and Hepatology, Sapporo Medical University School of Medicine, Sapporo 060-8543, Japan; aizenblue138@gmail.com (T.I.); awakamay.dem1@gmail.com (T.Y.); a08m081@gmail.com (T.H.); yoshi_yokoyamaa@yahoo.co.jp (Y.Y.); polestar100100@gmail.com (Y.H.); hirarin95@yahoo.co.jp (D.H.); waga_a05m@yahoo.co.jp (K.W.); 2 Department of Gastroenterology and Hepatology, Tokyo Medical University, Tokyo 160-0023, Japan; itoitakao@gmail.com

**Keywords:** autophagy, autophagy-related gene, mitophagy, cancer, neurodegenerative disease, cardiovascular, liver disease

## Abstract

Autophagy refers to the process involving the decomposition of intracellular components via lysosomes. Autophagy plays an important role in maintaining and regulating cell homeostasis by degrading intracellular components and providing degradation products to cells. In vivo, autophagy has been shown to be involved in the starvation response, intracellular quality control, early development, and cell differentiation. Recent studies have revealed that autophagy dysfunction is implicated in neurodegenerative diseases and tumorigenesis. In addition to the discovery of certain disease-causing autophagy-related mutations and elucidation of the pathogenesis of conditions resulting from the abnormal degradation of selective autophagy substrates, the activation of autophagy is essential for prolonging life and suppressing aging. This article provides a comprehensive review of the role of autophagy in health, physiological function, and autophagy-related disease.

## 1. Introduction

Autophagy is a general term for the physiological process by which cells direct their components to the lysosome via autophagosomes for degradation. In the 1950s, with the development of electron microscopy, Christian de Duve reported the existence of various hydrolases, leading to the discovery of lysosomes as cellular organelles [1]. In 1963, de Duve termed autophagy as a phenomenon during which cells fuse protein-containing vesicles with lysosomes, leading to the decomposition of cellular protein. The vesicles were named autophagosomes.

Autophagy is broadly divided into macroautophagy, chaperone-mediated autophagy (CMA), and microautophagy. The term autophagy often refers to macroautophagy.

Macroautophagy breaks down cargo, allowing for the recycling of the resulting macromolecules. Cytoplasmic macroautophagy substrates are confined within a transient double-membrane organelle known as the autophagosome, which then fuses with lysosomes and vacuoles. The recycling of substrates through macroautophagy plays an important role in maintaining cellular homeostasis [2].

CMA is a selective form of autophagy, which exclusively targets proteins for lysosomal degradation. The major role of this pathway in cellular metabolism was predicted to be the supply of free amino acids generated following protein degradation. However, recent studies have shown that impaired CMA significantly alters glucose and lipid metabolism and, consequently, the energy metabolism of the whole organism, while also playing an important role in the regulation of cellular metabolism in response to various nutrients [3]. Microautophagy is a non-selective lysosomal degradation process in which autophagic tubing mediates both cytoplasmic lumen entry and vesicle cleavage by direct engulfment of cytoplasmic cargo. It has a role in maintaining homeostasis and cell survival [4]. Further, autophagy mechanisms complement each other to degrade intracellular substances and supply degradation products, allowing for the maintenance of homeostasis in vivo.

Disruption of autophagy causes inhibition of ubiquitination, accumulation of reactive oxygen species (ROS), reduced mitochondrial function, and increased genomic instability, resulting in an overall decrease in the quality of intracellular components [5,6]. Thus, disruption of autophagy disturbs cellular homeostasis and contributes to the development of various diseases.

Recent studies reported that autophagy is involved not only in disease but also in aging and life span extension [7]. The activation of autophagy has been shown to be essential for lifespan extension in several model organisms. Additionally, autophagy function has been demonstrated to decline with age [8,9,10]. The current work provides an overview of the process and different types of autophagy as well as its involvement in numerous diseases.

## 2. Autophagy: Overview of Autophagy and Its Molecular Mechanism

Autophagy is induced by various reactions in the organism, such as amino acid starvation, decreased insulin levels, decreased ATP levels, and hypoxia. When autophagy is induced, the unc-51-like autophagy activating kinase 1 (ULK1)/autophagy-related gene (Atg)1 complex consisting of autophagy-related factors is recruited, followed by the formation of a membrane vesicle called the phagophore, which matures into a spherical lipid bilayer vesicle named as the autophagosome. The autophagosome then fuses with a lysosome or a vacuole to decompose the cytoplasmic components it has taken up [11]. The transcription factor EB (TFEB) is a member of the microphthalmia family of basic helix-loop-helix leucine-zipper (bHLH-Zip) transcription factors (MiT family). TFEB is a major regulator of the autophagy lysosomal pathway. TFEB regulates starvation-induced autophagy transcriptional regulation by driving the expression of autophagy and lysosomal genes [12]. Under cellular conditions that inactivate the mammalian target of rapamycin complex 1 (mTORC1) kinase complex, such as starvation, TFEB phosphorylation is repressed and allows TFEB to move into the nucleus. TFEB in the nucleus binds to the coordinated lysosomal expression and regulation (CLEAR) element of genes, thus inducing the expression of genes involved in processes, such as autophagy and lysosomal exocytosis [13,14,15]. The mTORC1 kinase complex is a serine/threonine protein kinase of the class III phosphatidylinositol 3-kinase (PI3K) associated kinase family that plays an inhibitory role in autophagy [16]. Specifically, by phosphorylating ULK1 and Atg13, mTORC1 negatively regulates the activity of the ULK1 complex, which is composed of four factors, including ULK1, Atg13, focal adhesion kinase family interacting protein (FIP) 200, and Atg101 [17]. Additionally, mTORC1 regulates autophagy by directing the localization of TFEB to lysosomes [18]. On the lysosome surface, mTORC1 is activated by the GTP binding form of the brain-enriched low-molecular-weight G protein Ras homolog (Rheb) [19]. The subcellular localization of mTORC1 is regulated by the low-molecular-weight G protein Rag. Rag consists of RagA/B and RagC/D heterodimers and is associated with the activity of mTORC1 by altering its conformation by binding to GDP/GTP under conditions of amino acid starvation [20]. Inactivation of mTORC1 mobilizes a PI3K complex downstream of ULK1 to form the phagophore. The class III PI3K complex contains multiple complexes with different constituent factors, and the complex consisting of vacuolar protein sorting (VPS) 34, VPS15, Beclin-1, and Atg14 functions in the early stages of phagophore formation. VPS34 of the PI3K complex generates phosphatidylinositol-3-phosphate (PI3P) [21], a process accompanied by the formation of omegasomes. Furthermore, the WD repeat domain, phosphoinositide interacting protein (WIPI), is mobilized as a PI3P effector protein, and WIPI2 acts on Atg2. Atg2 functions to connect the endoplasmic reticulum (ER) membrane and phagophore, has lipid transporting ability, and is considered to play an important role in supplying lipids to autophagosomes [22]. Two types of ubiquitin-like binding reaction systems, namely the microtubule-associated protein light chain 3 (LC3) binding reaction system and Atg12-Atg5 binding reaction system, are involved in forming autophagosomes. In the LC3-binding system, LC3, a ubiquitin-like protein, is involved in the elongation and closure of the autophagosome membrane by binding to phosphatidylethanolamine (PE). In the Atg12-Atg5-binding system, the ubiquitin-like protein Atg12 covalently binds to Atg5 via Atg7 and Atg10. The Atg12-Atg5-binding system then interacts and binds to Atg16L1 [23,24]. This conjugate is mobilized to the phagophore to produce E3-like activity toward the LC3-PE complex. The lipidized LC3, via its E3-like activity, can selectively degrade specific substrates by interacting with multiple selective autophagy receptors [25,26].

**Figure 1:**
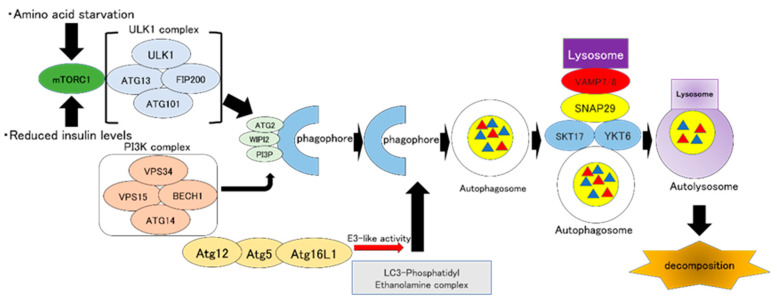
ULK1 complex involved in initiating autophagy is regulated by mTORC1. Amino acid starvation and reduced insulin levels lead to the inactivation of mTORC1, resulting in the induction of autophagy. The class III phosphatidylinositol 3-kinase (PtdIns3K) complex is mobilized downstream of the PI3K complex to form a phagophore. VPS34 of the PI3K complex forms PI3P, and WIPI is mobilized, and WIPI2 acts on Atg2. The Atg12-Atg5-Atg16L1 complex has E3-like activity against the LC3-PE complex on phagophores. Autophagosome formation is completed by the LC3-PE complex and Atg12-Atg5-Atg16L1 complex. Autophagosomes form SNAP29, and lysosome VAMP7/VAMP8 and SNARE conjugates by STX17 and YKT6 complexes, resulting in the fusion of autophagosomes and lysosomes. ULK1: Unc-51-like autophagy activating kinase 1, mTORC1: mammalian target of rapamycin complex 1, PI3K: Class III phosphatidylinositol 3-kinase, VPS34: phosphatidylinositol 3-kinase, PI3P: phosphatidylinositol-3-phosphate, WIPI: WD-repeat protein interacting with phosphoInositides, Atg: Autophagy-related gene, LC3: Microtubule-associated protein light chain 3, PE: Phosphatidylethanolamine, SNAP: Synaptosomal-associated protein, VAMP: Vesicle-associated membrane protein, SNARE: Soluble N-ethyl maleimide-sensitive protein (NSF) attachment protein receptor, STX: Syntaxin.

Autophagosome formation is completed by extension and closure of the phagophore. The endosomal sorting complex required for transport (ESCRT) complex is involved in autophagosome closure. Two types of soluble *N*-ethyl maleimide-sensitive protein (NSF) attachment protein receptor (SNARE) complexes form a synaptosomal-associated protein (SNAP)29, lysosomal vesicle-associated membrane protein (VAMP)7/VAMP8, and SNARE conjugates, enabling fusion of autophagosomes and lysosomes [27,28]. The fusion of lysosomes and autophagosomes results in the formation of autolysosomes, which degrade the cargo (Figure 1).

## 3. Autophagy and Health: Selective and Non-Selective Autophagy and Physiological Functions

Although autophagy was originally thought to be a non-selective degradation mechanism, more recent studies have shown that autophagy functions selectively under certain conditions. Non-selective autophagy randomly degrades cytoplasmic components, whereas, during selective autophagy, selectivity is determined by cargo labeling and adaptor proteins in the autophagosome [29]. Adaptor proteins are a group of proteins that bind to cargo and include the LC3 or gamma-aminobutyric acid A receptor-associated protein (GABARAP) family of autophagosome-localized proteins; moreover, cargoes are labeled by ubiquitination or localization of adaptor proteins to cargoes [29]. Typical autophagy receptors that define the selectivity of autophagy include p62/Sqstm1 (p62). p62 functions as an adapter between selective autophagy and ubiquitin signaling [30]. The expression of p62 is induced by TFEB [12]. When damaged mitochondria and invasive microbes emerge, p62 is phosphorylated and traps the microbes. Further phosphorylation of Ser349 (human)/Ser351 (mouse) by mTORC1 increases the affinity between p62 and Keap1. Phosphorylated p62 bound to Keap1 is degraded in the autophagy pathway by interacting with LC3 through the LC3 interacting region (LIR) [31,32]. The names of selective autophagy subtypes and a list of substrates to be degraded are listed in Table 1.

### 3.1. Mitophagy

Mitophagy is a process involving the selective degradation of impaired mitochondria. Mitochondria play important functions in the cell, such as supplying energy through ATP production, phospholipid biosynthesis, and apoptosis induction [33,34]. Mitochondria produce ROS via electrons leaking from the electron transport chain, which react with oxygen in the surrounding environment [33]. Accumulation of dysfunctional mitochondria and production of ROS are associated with tumorigenesis [33,35]. Therefore, mitophagy contributes to the suppression of tumor formation by efficiently removing aberrant mitochondria. In fact, mitophagy deficiency resulting from the loss of Bcl-2 adenovirus E1B 19 kDa-interacting protein 3 (BNip3), a hypoxia-inducible protein that targets mitochondria, results in accelerated mammary tumor progression [36]. The phosphatase and tensin homolog (PTEN)-induced kinase (PINK)1/parkin complex phosphorylates ubiquitin, acts as a mitochondrial identification marker during mitophagy, and, in its defective forms, is known to be a causative factor of hereditary Parkinson’s disease (PD) [37]. It has been reported that Parkin-deficient mice have lost the ability to efficiently differentiate adipocytes [38]. This suggests that mitophagy also plays a role in cell differentiation.

### 3.2. Allophagy

Mitochondrial DNA (mtDNA) is known to be maternally inherited in various organisms. However, the exact mechanism of mtDNA inheritance remains unclear. A recent study of *Caenorhabditis elegans* has revealed that post-fertilization, paternal organelles are labeled with ubiquitin and degraded after phosphorylation of the autophagy receptors ALLO-1 and IKKE-1 in the process of selective autophagy [39]. This phenomenon is known as allophagy [40]. Selective autophagy for paternal mitochondria has been observed not only in *C. elegans* but also in *Drosophila* and mice [41,42]. Therefore, it is considered important to understand the mechanism of maternal inheritance.

### 3.3. ER-Phagy

The ER is divided into rough and smooth-surfaced ER based on the presence or absence of surface ribosomes. It plays a role in protein folding, transport, lipid synthesis, drug metabolism, and calcium ion accumulation [43]. Particularly, it has long been suggested that autophagy is involved in the degradation of deformed ERs due to drug metabolism [44,45]. Khaminets et al. also showed that the FAM134 reticulon protein family is an ER-phagy receptor that binds to LC3 and GABARAP [46]. ER-phagy receptor FAM134B-deficient mice exhibit dilatation of the ER and develop peripheral neuropathy as a clinical phenotype [46]. Further, mutations or changes in FAM134B expression have been observed in various malignant tumors [47]. These data suggest the involvement of ER-phagy in peripheral nerve homeostasis and the development of malignancies.

### 3.4. Lysophagy

Lysosomes possess a variety of hydrolytic enzymes that play an important role in intracellular digestion. When lysosomes damage is limited, the cell can repair lysosomes by means of ESCRT machinery [48]. When this repair fails, lysosomes are tagged with ubiquitin to initiate their clearance by selective macroautophagy in a process termed as lysophagy [49]. Lysophagy is necessary for lysosome quality control. Hyperuricemia, type 2 diabetes mellitus, amyloid-beta protein, tau protein, and Huntingtin protein cause lysosomal damage, and there are reports that a decrease in lysophagy is associated with lifestyle-related diseases and neurodegenerative diseases [49].

### 3.5. Nucleophagy

The nucleus is one of the most important cellular organelles and is responsible for harboring the cell’s genetic material as well as the site of transcription. Nucleophagy occurs in both a macro- and micronucleophagic manner [50,51]. Particularly, the selective degradation of nuclear lamina by autophagy has been suggested to limit tumor formation [52].

### 3.6. Pexophagy

Peroxisomes are organelles involved in various metabolic activities, such as purine catabolism, long-chain fatty acid beta-oxidation, bile acid synthesis, and phospholipid synthesis. These autonomously replicating organelles generate ROS as a byproduct of fatty acid β-oxidation. Cells maintain peroxisome homeostasis through pexophagy, which inhibits peroxisome biosynthesis disorders, oxidative damage, and diseases, such as cancer [53]. Pexophagy is a catabolic process for the selective degradation of peroxisomes by autophagy. Recent studies have shown that peroxisome membrane proteins, such as PEX3 and PEX5, are important regulators of pexophagy [53,54].

### 3.7. Lipophagy

Lipid droplets are structures in which phospholipids cover the lipid ester mass, which is mainly composed of triglycerides and cholesterol. The accumulation and utilization of lipids in the cell is important for the maintenance of energy in the body. Under starvation conditions, cellular lipids stored as triglycerides in lipid droplets are hydrolyzed to fatty acids. Lipophagy is the process by which lipid droplets are selectively degraded [55]. Increased triglycerides and lipid droplets in Atg7-deficient mouse hepatocytes suggest an important role for lipophagy as a lipolytic system in vivo [55]. Takahashi et al. found that fat accumulation in the liver of Atg7-deficient mice occurred not in Atg7-deficient hepatocytes but rather in hepatocytes expressing differentiated Atg7 from oval cells [56]. This is an important finding, clarifying the relationship between fat metabolism and autophagy.

### 3.8. Xenophagy

Bacteria and viruses invade the cytoplasm of various cell types to multiply. The autophagosome recognizes intracellular pathogens as a foreign entity, isolates them, and then transports them to the lysosome for degradation and removal in a process known as xenophagy [57]. Xenophagy is triggered by ubiquitination following membrane damage during bacterial infection [58]. However, some bacteria, such as *Legionella pneumophilia*, inhibit autophagy by suppressing phagophore formation [59].

### 3.9. Aggrephagy

Despite the normal formation of the isolation membrane or autophagosome, abnormalities may occur at the stage of incorporation of its substrate molecules into the autophagosome, and abnormal autophagosomes and aggregates may accumulate in cells [60]. Abnormal protein aggregates cause impaired nerve membrane permeability, irregular calcium homeostasis, inflammation, oxidative stress-induced neurotoxicity, and physiological abnormalities [61,62]. The mechanism via which aggregated proteins are selectively processed by autophagy is termed as aggrephagy [63].

### 3.10. Ribophagy

In vivo, the ribosome functions as a site for protein synthesis by translating information encoded in mRNA into polypeptides. Kraft et al. reported that there is a selective autophagy pathway in which ribosomes are preferentially degraded in nitrogen-starved budding yeast; they named this pathway ribophagy [64]. Under starvation conditions, ribophagy effectively suppresses new protein synthesis and recycles nutrients. Wyant et al. found that nuclear fragile X mental retardation-interacting protein 1 (NUFIP1) is a receptor for ribosomal selective autophagy [65].

### 3.11. Nuclear Pore Complex (NPC)-Phagy

The nuclear pore complex (NPC) is a large protein assembly embedded in the nuclear envelope. The NPC mediates transport between the nucleus and cytoplasm and plays an important role in gene expression. It has remained unclear whether the protein complexes within the organelle can undergo selective autophagy. In 2020, two research groups have demonstrated that NPC is degraded via receptor-dependent selective autophagy [66,67]. NPC-phagy contributes to the overall NPC quality and quantity control. However, the role of NPC-phagy in disease remains unclear.

### 3.12. RN/DN-Autophagy

RN/DN-autophagy (RDA) is an autophagy pathway in which nucleic acids are directly transported across the lysosomal membrane and degraded within the lysosome [68,69]. In these pathways, RNA and DNA are taken up and degraded directly into the lysosome, respectively. lysosome-associated membrane protein (LAMP) 2C, a lysosomal membrane protein, binds to RNA/DNA and functions as a receptor [68,69]. SID1 transmembrane family, member 2 (SIDT2), a transmembrane protein, is found to function as a nucleic acid transporter on the lysosomal membrane in RDA [70]. Tan et al. suggested that SIDT2 and RNautophagy contributed to the development of pulmonary and gastrointestinal adenomas in mice [71]; however, the detailed mechanisms of RDA remain unclear.

## 4. Autophagy and Various Diseases

### 4.1. Liver Diseases

The liver is an important organ for protein synthesis in the body. Autophagy is essential for the maintenance of hepatocyte homeostasis and is deeply involved in various hepatic diseases.

Non-alcoholic fatty liver disease (NAFLD) is caused by overnutrition and is often associated with metabolic diseases, such as diabetes mellitus and dyslipidemia. In NAFLD, decreased autophagy in hepatocytes causes hepatotoxicity and accumulation of lipid droplets, both of which contribute to hepatocyte death. mTORC1, which regulates the autophagy-starting complex, is activated by overfeeding and, consequently, suppresses autophagy [72]. Hyperinsulinemia caused by overnutrition also activates mTORC1 via the insulin receptor substrate 1 (IRS1)-PI3K-Akt/ protein kinase B (PKB) pathway [73], which negatively regulates autophagy. The presence of Rubicon protein, which inhibits autophagosome and lysosome fusion, is also thought to be involved in the pathogenesis of NAFLD. In hepatocyte-specific Rubicon protein knockout mice, autophagy suppression is abolished after feeding a high-fat diet, and hepatotoxicity, accumulation of fatty droplets, and fatty liver are improved [74]. In addition, the expression of Rubicon is increased in cultured hepatocytes after treatment with palmitic acid and in NAFLD [74]. These results suggest that the pathophysiology of NAFLD is related to the suppression of autophagy via delayed degradation of Rubicon.

Autophagy has also been observed in alcohol-induced liver injury. Experiments in acute alcoholic hepatitis model mice and cultured liver cells have revealed that ROS is generated during ethanol metabolism, whereas mTOR is inhibited. As a result, autophagy is enhanced and selectively targets mitochondria damaged by ethanol as well as lipid droplets, thus protecting the liver from ethanol-induced toxicity [75]. It has been reported that autophagy is impaired at the lysosome in a long-term alcoholic liver injury mouse model [76].

### 4.2. Diabetes

Diabetes mellitus is a metabolic disease characterized by defective insulin secretion and action, which results in hyperglycemia and leads to systemic disorders. Diabetes mellitus is classified into type 2 diabetes mellitus, which develops because of inadequate insulin secretion and resistance, and type 1 diabetes mellitus, which occurs as a result of insulin depletion caused by the destruction of pancreatic β-cells by the body’s own immune system. In pancreatic cell-specific Atg7 knockout mice, the number of β-cells has decreased because of increased β-cell apoptosis and decreased β-cell proliferative capacity. This has resulted in decreased serum insulin levels and, consequently, impaired glucose tolerance [77]. Further, ubiquitinated protein aggregates localized to p62 proteins with both ubiquitin-binding and LC3-binding domains are accompanied by morphological changes in the mitochondria, ER, and vacuoles [77]. When a high-fat diet has been fed to control mice and pancreatic cell-specific Atg7 knockout mice, the autophagy of pancreatic β-cells has stimulated, and their number has increased in controls, whereas autophagy-deficient mice have exhibited no increase in the number of β-cells [78]. These results suggest that disruption of autophagy in pancreatic β-cells causes abnormalities in the turnover and function of cellular organelles, resulting in insulin deficiency and hyperglycemia [77,78]. In addition, ER function is important for the stimulation and insulin secretion of β-cells. Further, β-cells are constantly exposed to ER stress. ER stress is considered to contribute to the development of human islet amyloid polypeptide (hIAPP), a peptide involved in blood glucose regulation [79], which forms amyloid aggregates associated with β-cell death and the development of type 2 diabetes [79]. Blocking autophagy in pancreatic β-cells has increased the toxicity of hIAPP [79], and mice expressing hIAPP in autophagy-deficient β-cells have developed diabetes, whereas hIAPP or autophagy deficiency alone has not induced diabetes [80]. These results suggest that autophagy plays an important role in eliminating hIAPP toxicity in the pancreas and, thus, in preventing the development of diabetes.

### 4.3. Kidney Diseases

The kidneys excrete unwanted products from the body by filtering blood in the glomerulus while reabsorbing minerals, microproteins, and water in the tubules. Proximal tubule-specific autophagy-deficient mice have accumulated deformed mitochondria and cytoplasmic inclusions, resulting in cellular hypertrophy and degeneration, which have not been observed in wild-type controls [81]. Autophagy-deficient mice have exhibited increased apoptosis of proximal tubular cells as well as an accumulation of p62 and ubiquitin-positive cytoplasmic inclusions, resulting in significantly elevated serum levels of urea nitrogen and creatinine [81].

Acute renal injury is a disorder of internal homeostasis and waste excretion due to tubular dysfunction caused by infection, drug exposure, or ischemia; the proximal tubular tissue is particularly susceptible to the aforementioned stimuli [81,82,83,84]. Enhanced autophagy in proximal tubular cells has been observed in mice with nephrotoxicity induced by cisplatin, an anticancer drug [85]. Administration of chloroquine, which suppresses autophagy, to a mouse model of cisplatin-induced renal injury has resulted in an increase in the extent of damage to most tubules, severe injury, and massive tubular lysis [85]. In a mouse model of cisplatin-induced kidney injury, PINK1/parkin gene expression, which is related to mitophagy, has increased in renal tissues, and the mTOR inhibitor rapamycin has mitigated the onset of cisplatin nephrotoxicity by stimulating autophagy and mitophagy [86]. PINK1/parkin knockout mice do not show mitophagy in the kidneys, and cisplatin treatment has caused more severe renal dysfunction and tissue damage [86]. These findings suggest that the activation of autophagy and mitophagy in the proximal tubules plays a cytoprotective role in acute kidney injury.

Chronic kidney disease (CKD) is a long-term impairment of renal function caused by diabetes mellitus, hypertension, and chronic nephritis, among other causes. The glomerular wall for blood filtration consists of three layers as follows: glomerular endothelial cells, glomerular basement membrane, and podocytes. Autophagy in podocytes is particularly important for maintaining renal function [87], and impaired podocyte autophagy may be associated with diabetic nephropathy and age-related deterioration of renal function [87,88]. Podocyte autophagy suppression in mice through podocyte-specific deletion of Atg5 has caused glomerulopathy with the accumulation of oxidized and ubiquitinated proteins, ER stress, and proteinuria, resulting in podocyte loss and delayed glomerulosclerosis [87].

Autophagy activity is required for the maintenance of podocyte function. However, as observed in in vivo diabetes models and high glucose conditions in vitro, podocyte autophagy is suppressed, leading to podocyte dysfunction [88].

Autophagy in podocyte cells is involved in age-related CKD pathophysiology. Podocyte-specific Atg5-deficient mice exhibit worsening of proteinuria with age [87]. Further, proximal tubule-specific Atg5-deficient mice show age-related renal dysfunction [81]. These findings indicate that autophagy functions in a protective manner against renal senescence.

### 4.4. Heart Diseases

Autophagy plays an important role in the quality control of cellular organelles to maintain cardiac function [89]. Particularly, mitochondria in the myocardium produce ATP, which is necessary for cardiac pulsation, and mitochondrial impairment leads to cardiac dysfunction [90].

Cardiac-specific Atg5-deficient mice have exhibited accumulation of ubiquitinated proteins as well as increased ER stress, and pathological analysis has revealed a disorganized sarcomere structure, mitochondrial misalignment and aggregation, and apoptotic cardiomyocyte death in Atg5-deficient hearts [89]. The size and contractility of the left ventricle in cardiac-specific Atg5-deficient mice are comparable to those of wild-type mice at 3 months of age. However, knockout mice have experienced heart failure at 6 months of age. The knockout mice have also exhibited a significant increase in left ventricle size and a decrease in left ventricle fractional shortening at 10 months age compared with wild-type mice [91]. In a pressure-loaded mouse model of aortic stenosis and heart failure, autophagy and mitophagy are activated. However, their activation is transient, and, over time, autophagy and mitophagy become inactive with the reduction of mitochondrial and cardiac function [69]. Tat-Beclin-1, an autophagy-inducing peptide, has been reported to reduce mitochondrial dysfunction and the progression of heart failure by increasing autophagy and mitophagy [92]. In patients with dilated cardiomyopathy, autophagy in cardiomyocytes is positively correlated with the prognosis of heart failure [93]. In vitro and in vivo, the pro-apoptotic kinase Mst1 inhibits autophagy. In contrast, inhibition of Mst1 enhances autophagy and prevents the progression to heart failure following myocardial infarction [94].

These findings suggest that autophagy and mitophagy contribute to the maintenance of cardiac function and its improvement after the onset of heart disease.

### 4.5. Inflammatory Bowel Disease

Inflammatory bowel disease (IBD) is an intractable chronic disease caused by a complex mixture of genetic predisposition, alterations in the intestinal microbiota due to dietary changes, and an abnormal increase in intestinal inflammation, among other factors.

A genome-wide association analysis conducted in 2007 identified *Atg16L1* as a Crohn’s disease (CD) susceptibility gene [95], and the relationship between IBD and genes associated with autophagy, such as nucleotide-binding oligomerization domain-containing protein (NOD)2, immunity-related GTPase family M protein (IRGM), leucine-rich repeat kinase (LRRK)2, and ULK1, has also been examined [96,97,98,99,100]. It is well-established that autophagy is crucial for the maintenance of intestinal epithelial cell function, regulation of intestinal physiology, the intestinal immune response, and maintenance of the intestinal microbiota.

Paneth cells produce several antimicrobial peptides, such as α-defensins, to prevent bacterial invasion. Goblet cells produce mucus to prevent the invasion of foreign entities [101,102]. Intestinal mucosal permeability is increased in IBD. Nighot et al. reported that autophagy modulates intracellular permeability and the strength of the tight junction barrier [103]. *NOD2*, located on chromosome 16q12.1, encodes a pattern recognition receptor associated with intestinal immunity. Normally, it recruits autophagy protein Atg16L1 to the plasma membrane. However, mutant NOD2 fails to recruit Atg16L1 to the plasma membrane, impairing the defensive function of the autophagosome.

Therefore, NOD2 mutants exhibit autophagy defects, and this process is thought to be related to CD [104,105]. It has been reported that mutant Atg16L1 is destabilized by caspase 3 under stress conditions, such as starvation, resulting in reduced autophagy activity, reduced antibacterial activity, and increased proinflammatory cytokine production [106]. Atg5- and Atg7-deficient mice have exhibited Paneth cell morphological abnormalities [107]. IRGM belongs to the interferon-inducible GTPases family, has a strong bactericidal effect against intracellular pathogens, and regulates the inflammasome, thus increasing IL-1β production [108,109]. *LRRK2*, originally identified as a causative gene for familial Parkinson’s disease, has also been identified as a susceptibility gene for CD [110,111]. *LRRK2* knockout mice have an altered gut microbiota, leading to an impaired defense against infection and an increased risk of infection. ULK1 causes a lack of lysozyme in Paneth cells [112].

### 4.6. Neurodegenerative Diseases

Numerous neurodegenerative diseases are caused by autophagy deficiency in neurons associated with the mutation or deletion of autophagy-related genes. Autophagy-related neurodegenerative diseases are abundant and diverse (Table 2). In this review, we have focused on PD and static encephalopathy of childhood with neurodegeneration in adulthood (SENDA)/β-propeller protein-associated neurodegeneration (BPAN) as an inherited disease. PD is a symptom-based disease caused by degenerated dopaminergic neurons in the mesencephalic nerve system. Parkin, a ubiquitin ligase encoded by *PARK2*, and PINK1, encoded by *PARK6*, work together on the outer mitochondrial membrane to induce mitophagy [113,114,115,116,117]. In fact, mutated Parkin and PINK1 compromise mitophagy, resulting in stagnation of mitochondrial metabolism and accumulation of ROS [118]. Therefore, this pathway is considered to be closely related to the development of PD.

SENDA/BPAN is characterized by mental developmental delay, epileptic seizures, and decreased motor function from childhood; in adulthood, peronism and dementia develop. Autophagy-related Wdr45 has been identified as a causative gene [126,127]. Neurospecific Wdr45 knockout mice have shown decreased brain function, reduced autophagic activity in the brain, and decreased protein resolution [128]. Wan et al. showed that induction of autophagy or reduction of ER stress in Wdr45 knockout mice resulted in the avoidance of cellular apoptosis [119]. This phenomenon suggests that activation of autophagy is a promising help for treating this disease.

### 4.7. Cancer

Disruption of autophagic cell quality control can lead to tumor development. Tumor growth is also related to cell nutrient supply through the activation of autophagy. Hence, both inhibition and activation of autophagy contribute to tumor development and growth via different pathways.

Atg5 mosaic-deficient mice and liver-specific Atg7-deficient mice form benign tumors in the liver [129,130]. The selective autophagy protein p62 binds to ubiquitinated proteins, after which it is subjected to autophagy and degraded [131]. In autophagy-deficient mice, p62 markedly accumulates and binds to Keap1, an adaptor protein of Cullin3-type ubiquitin ligase, to inhibit the binding of Keap1 to transcription factor NF-E2-related factor (Nrf) 2 [32]. As a result, degradation of Nrf2 is inhibited, and tumor growth is promoted [32]. In addition, liver tumors caused by autophagy suppression are greatly reduced following the simultaneous loss of p62 and Nrf2 [132,133]. Further, local amplification of p62 on chromosome 5q occurs in renal cancer [134]. p62 regulates not only Nrf2 but also mTOR and nuclear factor-kappa B (NFκB), all of which play important roles in the signaling pathways of cancer development [135]. Thus, p62 may play a role in the mechanism of cancer control by autophagy.

Autophagy contributes to the growth of advanced lung cancer, pancreatic ductal adenocarcinoma (PDAC), melanoma, and breast cancer in various mouse models with autophagy gene deletions or RAS/BRAF mutations [136,137,138,139,140,141,142]. Autophagic activity is particularly strong in PDAC, and inhibition of autophagy inhibits tumor growth [138]. Microphthalmia/transcription factor E (MiT-TFE) family transcription factors are a series of inducers of autophagy/lysosome pathway-related gene expression [143]. The interaction between importin8 and importin7, which is responsible for the nuclear transfer of MiT-TFE family members in PDAC, is enhanced, and the MiT-TFE family is permanently transferred into the nucleus [144]. Normally, autophagy requires dephosphorylation of ULK1 by inactivation of mTORC1, and protein phosphatase 2A (PP2A) has been identified as a phosphatase of ULK1 [145]. Further, PP2A enzyme activity is upregulated, and autophagy activity is enhanced in PDAC cell lines [145]. Acute suppression of KRAS and inhibition of extracellular signal-regulated kinase (ERK) has resulted in greater autophagy fluxes when KRAS is acutely suppressed in a panel of human and mouse PDAC cell lines using siRNA and small molecule inhibitors of the KRAS effector, ERK mitogen-activated protein kinase (MAPK). This result shows that both autophagy and ERK MAPK are largely responsible for the PDAC activity [146].

Autophagy is also intimately involved in the development and progression of non-small cell lung cancer (NSCLC). Deletion of Atg7 in a mouse model of NSCLC has resulted in the accumulation of abnormal mitochondria and inhibition of tumor cell growth [147,148]. This result indicates the importance of normal mitochondrial function for NSCLC proliferation and activation. In addition, the expression of LAMP2A, a major receptor protein for CMA, has been upregulated in NSCLC cell lines and patient tumors, resulting in decreased survival and platinum resistance in a patient with NSCLC [149]. LAMP2A knockdown has suppressed tumorigenicity and sensitized tumors to cisplatin treatment in mice with NSCLC. These results demonstrate that chemotherapy for NSCLC, targeting CMA, may be effective [149].

In colorectal cancer (CRC)-derived cell lines, p53 has stabilized autophagic activity by promoting LC3 degradation [150]. In addition, the autophagosome marker LC3-II protein has been found to be overexpressed in advanced CRC compared to in normal surrounding tissues [151]. LC3 expression levels may reveal the involvement of autophagy in cancer. Cho et al. found that the expression level of Atg5 in CRC was downregulated. However, immunohistochemical analysis of Atg5 expression in patients with CRC showed a correlation between lymph node infiltration and Atg5 expression [152]. Moreover, Ahn et al. showed that 95% of CRCs expressed Beclin-1 higher than in normal tissue, suggesting the contribution of Beclin-1 expression to both CRC and gastric cancer. However, there was no significant association of Beclin-1 expression with pathologic characteristics, including invasion, metastasis, and stage of CRC [153]. These observations indicate that Beclin-1 plays a role in CRC initiation but not in cancer progression. The high Beclin-1 expression has been correlated with overall survival and disease-free survival and served as a good independent prognostic marker in CRC [154], whereas high Beclin-1 expression has also been associated with poorer survival of patients with CRC treated with adjuvant 5-fluorouracil [155]. This study suggests that increased autophagy plays a role in the initiation of CRC as well as having a role in resistance to chemotherapy for CRC. Thus, the role of autophagy in CRC is still uncertain, and further research is required to elucidate how autophagy works to the initiation and development of CRC.

Autophagy is also associated with breast cancer. Liang et al. noted that Beclin-1 protein expression is often low in human breast epithelial carcinoma cell lines and tissues, but it is expressed at high levels and ubiquitously in normal breast epithelium. Thus, the reduced expression of autophagy proteins contributes to the initiation and progression of breast and other human malignancies [156]. In addition, Wei et al. found that the FIP200 deletion, which led to the inability to form autophagosome, suppressed mammary tumor initiation and progression in a mouse model of breast cancer [142]. In addition, inhibition of autophagy in dormant breast cancer cells has significantly reduced cell survival and metastatic burden in vivo [157], and inhibition of autophagy flux in dormant breast cancer cells has led to the accumulation of damaged mitochondria and ROS, resulting in cell apoptosis [157]. These studies suggest that autophagy is essential to the initiation and progression of breast cancer.

## 5. Conclusions

**Figure 2:**
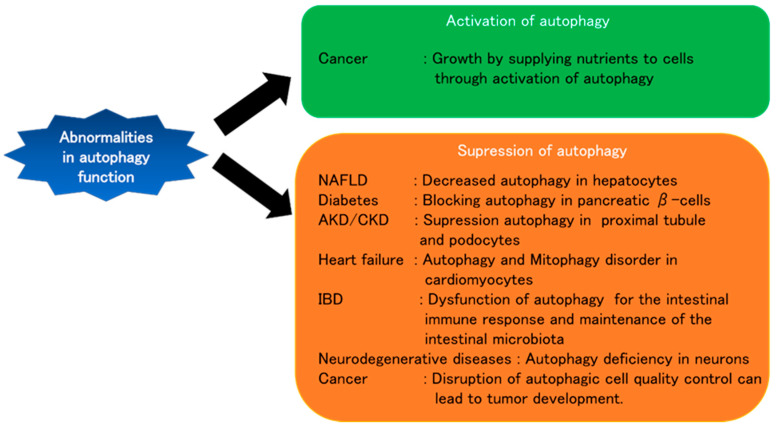
Number of publications have demonstrated that suppression of autophagy has detrimental effects on the maintenance of function in vivo. NAFLD, Diabetes, AKD/CKD, Heart failure, IBD, and Neurodegenerative diseases are associated with impaired autophagy function. However, in cancer, not only the suppression but also the enhancement of autophagy plays a major role in the development of the disease through different pathways. NAFLD: Non-alcoholic fatty liver disease, AKD: Akute kidney diseases and disorders, CKD: Chronic kidney disease, IBD: Inflammatory bowel disease.

The discovery of autophagy mechanisms, autophagy-related genes, and selective autophagy has not only played a major role in elucidating physiological processes but has also helped to reveal the pathophysiology of various diseases that had remained unclear. In addition to the suppression of autophagy, the enhancement of autophagy may also be detrimental to the maintenance of autophagic functions (Figure 2). Currently, various clinical trials are underway to provide better treatments for chronic diseases and cancer by modulating autophagy. We have summarized a selection of them in the following list (Table 3).

Further studies should be performed to identify substrate recognition adaptors of macroautophagy to determine more selective approaches for intervening in autophagy. From a more fundamental perspective, three-dimensional microscopy should be conducted to analyze autophagosomes, which will improve the understanding of the autophagic process. Although many autophagy genes have been identified in various studies, how they induce and execute autophagy is unclear. Advancement in the analysis of autophagy substrates and adapters will lead to the discovery of disease-specific autophagy-related drugs.

## Figures and Tables

**Table 1:** List of selective autophagy types and target cargo.

Name	Target Cargo
Mitophagy	Mitochondria
Allophagy	Paternal organelles
ER-phagy	Endoplasmic reticulum
Lysophagy	Lysosome
Nucleophagy	Nucleus
Pexophagy	Peroxisomes
Lipophagy	Lipid droplets
Xenophagy	Cellular pathogens
Aggrephagy	Abnormal protein aggregates
Ribophagy	Ribosomes
NPC-phagy	NPC
RN/DN-autophagy	RNA/DNA

NPC: Nuclear pore complex.

**Table 2:** Autophagy-related neurodegenerative disease.

Name	Mutated Gene	Mechanism	Reference
Parkinson’s disease	Parkin, PINK1	Mutated Parkin and PINK1 inhibit mitophagy	[118]
Static encephalopathy of childhood with neurodegeneration in adulthood β-propeller protein-associated neurodegeneration	Wdr45	Abnormalities in Wdr45 lead to impaired lysosomal degradation of ER proteins	[119]
Huntington’s disease	HTT	PolyQ extension in HTT reduces autophagy by acting on Beclin-1	[120]
Amyotrophic lateral sclerosis	OPTN1	ALS mutations interfere with efficient Parkin-mediated mitophagy degradation	[121]
Hereditary spastic paraplegia	ZFYVE26	ZFYVE26 mutation contributes to a defect in the fusion of autophagosomes and endosomes	[122]
Charcot–Marie–Tooth disease	RAB7A	RAB7A mutants reduce autophagic flux in HeLa cells	[123]
Frontotemporal dementia	SQSTM1	Mutations in SQSTM1 impair ubiquitin-mediated autophagic degradation	[124]
Alzheimer’s disease	APP	Mutant APP and amyloid β cause autophagy/mitophagy abnormalities in hippocampal neurons	[125]

PINK: Phosphatase and Tensin Homolog (PTEN)-induced kinase, ER: endoplasmic reticulum, PolyQ: polyglutamine, ALS: Amyotrophic lateral sclerosis.

**Table 3:** List of clinical trials for autophagy-related diseases.

Trial	Disease	NCT Number
Study of Autophagy and the Effects of GALIG Gene Products in HIV-1-Infected Patients Who Are Under Antiretroviral Therapy Since Primary Infection, Chronic Phase, or Never Treated	HIV Infections	NCT04160455
MEK and Autophagy Inhibition in Metastatic/Locally Advanced, Unresectable Neuroblastoma RAS (NRAS) Melanoma (CHLOROTRAMMEL)	Melanoma	NCT03979651
Study of Combination Therapy with the MEK Inhibitor, Cobimetinib, Immune Checkpoint Blockade, Atezolizumab, and the AUTOphagy Inhibitor, Hydroxychloroquine, in KRAS-mutated Advanced Malignancies	Gastrointestinal Cancer	NCT04214418
Sirolimus or Vorinostat and Hydroxychloroquine in Advanced Cancer	Advanced Cancers	NCT01266057
Akt Inhibitor MK2206 and Hydroxychloroquine in Treating Patients with Advanced Solid Tumors, Melanoma, Prostate, or Kidney Cancer	Advanced Malignant Solid Neoplasm Stage III Cutaneous Melanoma Stage III Prostate Cancer	NCT01480154
MLN9708 and Vorinostat in Patients with Advanced p53 Mutant Malignancies	Advanced Cancers	NCT02042989
LY3214996 +/− HCQ in Pancreatic Cancer	Pancreatic Cancer Advanced Cancer	NCT04386057
Combined Carfilzomib and Hydroxychloroquine in Patients with Relapsed/Refractory Multiple Myeloma	Multiple Myeloma	NCT04163107
Catalyzing the Containment of COVID-19	COVID-19	NCT04523090

GALIG: Galectin-3 internal gene, HIV: Human immunodeficiency virus, MEK: Mitogen-activated extracellular signal-regulated kinase, HCQ: Hydroxychloroquine, COVID-19: coronavirus disease 2019.
